# Supplementary material for: Patterns of Intron Gain and Loss in Fungi
Source: PLoS Biol. 2004 Nov 30;2(12):e422. doi: 10.1371/journal.pbio.0020422 (PMC532390; doi:10.1371/journal.pbio.0020422)
Supplement: Table S1 — Also available at http://genes.mit.edu/NielsenEtAl/. (4.3 MB ZIP). [file pbio.0020422.st001.zip › NielsenEtAl/html/846.html]

AN3063.1.NCU02401.1.MG03574.1.FG09837.1


```
 CLUSTAL W (1.82) Multiple Sequence Alignments - Introns Inserted


Sequence 1: MG03574.1	656 aa
Sequence 2: FG09837.1	634 aa
Sequence 3: NCU02401.1	707 aa
Sequence 4: AN3063.1	611 aa
Alignment Length: 756 aa
Number Identitical Residues: 189 aa
Alignment Score (without introns) 10656


MG03574.1 	MSANESGKGKGVPLRPANPAAVDAITNGTKADNVPQNESASFGPDSLPENCVEYMIFLID
NCU02401.1	-------------------------MESTNEPRPGDDAFNGFQ-MSLPENCVEYMLFLIN
FG09837.1 	-----------MSFRSA------IIEEEEISQKSGQTEETEFK-NHLPDNCVEYLLFYLD
AN3063.1  	-----------------------MAPMSQEDIEWFKSTFRPIPKPELPDDCVEYSLHYIS
          	                                .  .     :    **::**** :. :.

MG03574.1 	QQ-----LEPN-NVLSSLEAVKKAGVQLMESLAKDYIWQRDGFSLEIKSHAG1LLHLHGV
NCU02401.1	DQ-----PEAQRTTLSSLETIRKAALQRLDELTKDYIWQREALKLETRIENG1LVYLHGR
FG09837.1 	PQ-----LDAR-KQLSQVENIRKSAIDLANTLTKDYIWQKDEFNLALKNESG1LLYLHGI
AN3063.1  	SNPAPALVDEATDTRARLTEVQKSAAELSKQLLKDYIWQREAFRLEATKKDG1TTILSGR
          	 :.:.:  :      : :  ::*:. :  . * ******:: : *    . *    * * 

MG03574.1 	TDHGDSVEDEWLIVYVLRELTKSFNNIWIRVFDSDGEFLLVEAANVAPKWLNPEMDGNR~
NCU02401.1	TDYGDNVEDEWLIVYFLRTLSFSFPNLWIRIFDSDGEFLLIEAAKVLPKWLSPEMDANR~
FG09837.1 	TDYSDAVEDEWLIVYILRELSKSHPSLWIRVFDTDGEFLLVEAANVLPRWLNPEIDNNR~
AN3063.1  	TNFGDSVEDEWVIVYLLRELTKKHKDIWATVTDNDGQFLLAEAAGALPSWLEPEVADNR0
          	*:..* *****:***.** *: .. .:*  : *.**:*** *** . * **.**:  ** 

MG03574.1 	VWIHQGKLMLVPLAAGDNGASKS------ISLPDAVAFIKSQESSLVHSSFVESEAFYRL
NCU02401.1	VWINRGQLRIIPLLPSSSSSSPSSSKAHQISLHEALYVIRTTPNILIHSPLIEAEAFYRL
FG09837.1 	TWINTGHLLLVPVKDEDGLKTRN------LELPEAVEIIRAKREALVHSGFVEAEAFYRL
AN3063.1  	VWIHQGDLAIIKPKNEKRSRVTET-----ISLLEARSIIKDEPNRLMHSTMIQEEAFYRL
          	.**: *.* ::     .     .:     :.* :*  .*:   . *:** ::: ******

MG03574.1 	EKYPRQITDSIHCSLVTIPRKLAYVIHDRPKAIAPAVEAFYLRDPKALKPLLTDPPSREL
NCU02401.1	EKYPDQITSSLHHALVTIPRKLAYILHKRPKAVAPAIEAFYLRDPISLKPLLS-ATPDSL
FG09837.1 	EKYPAQISDSIHHSLVTIPRKLAHVLHSLPKSVAPAVETFYLRDPIALKRVIS--PSGPP
AN3063.1  	RNYPKQISENMHSALVIIPRKVAFLLHQKPAYISPAVEAFYLRDPIALRPLRS-KNTDDL
          	.:** **:..:* :** ****:*.::*. *  ::**:*:****** :*: : :   .   

MG03574.1 	EFPPVDLVTVSVRFTKVLFAQLRSQRFEPPPPAWAELLPPLPSESDVGAEPH--------
NCU02401.1	LFPPQDLVTISVRLTKVLYAQLKSQHFDPPLVWREHFVQTAQVQELSASETEGEARQQAL
FG09837.1 	VFPPQDLITTSVRFSKVLFAQLRSQRFDTPP-RWADVLRKVQKEVSSG-ESD--------
AN3063.1  	IFRPEDFVTVSTRFTRTGYAQVKSQDFPAPKSWLNKLPSTEDR-----------------
          	 * * *::* *.*:::. :**::** * .*     ..                       

MG03574.1 	---SARAQLEMGMKVTTGFELLAAGAEKSRSRVAREVAIVLDDLREDG-ADALPSDADIR
NCU02401.1	QKLQTLARLEIGMKLTTGFEILARGAEKSDSRVAREVALLLEDLKEDGEEAALPTDEEIK
FG09837.1 	---KVMARLDIGMKLTCGFEMLAAKADKSNNRVVRELAIVIEDLEEDG-IDALPSDEEIK
AN3063.1  	---KVYDRADTGMKLTCGFEMLLSDPQNQDKALVREMKLILEDLETGD--ESLPTNEEIQ
          	   ..  : : ***:* ***:*   .::. . :.**: ::::**. ..   :**:: :*:

MG03574.1 	AWKDVDRDDDESWLDINYDDFDRELDG----------------KKRAGAAGDSGKRDAGF
NCU02401.1	KWKDVDREDDDSWMDIDFADFERELAGGGGVQGESEQQHQRQQNKAGEGRDTAGHAVPTF
FG09837.1 	SWKDAERDDSEAWMDIDYQEFERELDG----------------KR----GNNTSNAKPGF
AN3063.1  	TWD--KREDDEKWLDISFEDLDTELKG-----------------KDKGKGAEKPQSAGGF
          	 *.  .*:*.: *:**.: ::: ** *                 :         :    *

MG03574.1 	GATNAQADLRKIVERFEAFLNDDDAGIDGAE-VDDMDMDDDEDD----DSDEDSDGEDKE
NCU02401.1	GDATTQADLRKIVSRFEAFLNDETAGLDGAE-IDEMDQDDDDDDLEDSDVDMDSEDEDKE
FG09837.1 	GDANTQSDLRKIVSRFEAFLNDDSAGMDGAE-LDEMDYDDEDDD----EFDDDSESEDKE
AN3063.1  	GDANAQENLQRIVAQFEKFLNDDSAGFEGADFIDDFETDSDIEE--DEEEDYGSEGEDRE
          	* :.:* :*::** :** ****: **::**: :*::: *.: ::  ..: * .*:.**:*

MG03574.1 	VSFDEEEFARMMREMMGLPATDRT---------------GKCPARPAAPPKG--------
NCU02401.1	VSFDEEQFSKMMREMMGIPTTTTTTAATTSSPPSSTTGPESAPRQPGTKGNGKGKEKVSF
FG09837.1 	VSFDEEEFAKMMREMMGLPSTDSN---------------NNTPKKTKHKSES--------
AN3063.1  	ASFDEEEFSRMMKEMMGMPSGPLMP------------PLNKPPKSSKAVNR--------V
          	.*****:*::**:****:*:    .               . *  .    .         

MG03574.1 	SAAAGEDEQE--------IKAIQELAAQMEAELNVHGALKLDPTPKKLAALKGKAPARGA
NCU02401.1	TVNDDKDEDKSMDTGHSEQEELEALMAQMEKELNSYGALQLDPQKAKTRSARALKGGVHE
FG09837.1 	PVMDEEDEE------------IQKLTTEFEAELNEHGALKLDPAAEKQPCLKNISQKEGE
AN3063.1  	EELDTDSEDD--------TEQIQQLASEMEAELKGTGVLDLNRPSDVSNDKRAVTRGTIE
          	     ..*:.         . :: * :::* **:  *.*.*:         :        

MG03574.1 	SSSSKMPVENLDS----DDDDEDDEDGEDG--ELNIDYNLAKNLLESFKSQGGMAGPAGN
NCU02401.1	ASSVQEIVKTAGGGRSDDDDDMNDEEGEEEGDEVGVDYNLAKNLLESFKSQAGMAGPTGN
FG09837.1 	TS-----LPNIE--------EEGGEDSEE---EVDIDYNLAKNLLESFKSQGGMAGPTGN
AN3063.1  	EAN-----------------SDPESDSED------VNINLVKNLLESLQGQSGTAGPAGN
          	 :.                 .   .:.*:      :: **.******::.*.* ***:**

MG03574.1 	MLGMMGMGLPRDEDDGRDGK------------------
NCU02401.1	LLGLMGMTLPRDEEDSDGEGKS----------------
FG09837.1 	LLGMMGFQLPRDEDEENDMNDEDEAGDSSSKGKRKAKA
AN3063.1  	MLSMMKLPMPKDDRPR----------------------
          	:*.:* : :*:*:
```
